# Distribution of geographical scale, data aggregation unit and period in the correlation analysis between temperature and incidence of HFRS in mainland China: A systematic review of 27 ecological studies

**DOI:** 10.1371/journal.pntd.0007688

**Published:** 2019-08-19

**Authors:** Xing-Hua Bai, Cheng Peng, Tao Jiang, Zhu-Min Hu, De-Sheng Huang, Peng Guan

**Affiliations:** 1 Department of Epidemiology, School of Public Health, China Medical University, Shenyang, China; 2 Department of Radiation Oncology, The First Affiliated Hospital of China Medical University, Shenyang, China; 3 Department of Mathematics, School of Fundamental Sciences, China Medical University, Shenyang, China; NIAID Integrated Research Facility, UNITED STATES

## Abstract

**Background:**

Changes in climate and environmental conditions could be the driving factors for the transmission of hantavirus. Thus, a thorough collection and analysis of data related to the epidemic status of hemorrhagic fever with renal syndrome (HFRS) and the association between HFRS incidence and meteorological factors, such as air temperature, is necessary for the disease control and prevention.

**Methods:**

Journal articles and theses in both English and Chinese from Jan 2014 to Feb 2019 were identified from PubMed, Web of Science, Chinese National Knowledge Infrastructure, Wanfang Data and VIP Info. All identified studies were subject to the six criteria established to ensure the consistency with research objectives, (i) they provided the data of the incidence of HFRS in mainland China; (ii) they provided the type of air temperature indexes; (iii) they indicated the underlying geographical scale information, temporal data aggregation unit, and the data sources; (iv) they provided the statistical analysis method that had been used; (v) from peer-reviewed journals or dissertation; (vi) the time range for the inclusion of data exceeded two consecutive calendar years.

**Results:**

A total of 27 publications were included in the systematic review, among them, the correlation between HFRS activity and air temperature was explored in 12 provinces and autonomous regions and also at national level. The study period ranged from 3 years to 54 years with a median of 10 years, 70.4% of the studies were based on the monthly HFRS incidence data, 21 studies considered the lagged effect of air temperature factors on the HFRS activity and the longest lag period considered in the included studies was 34 weeks. The correlation between HFRS activity and air temperature varied widely, and the effect of temperature on the HFRS epidemic was seasonal.

**Conclusions:**

The present systematic review described the heterogeneity of geographical scale, data aggregation unit and study period chosen in the ecological studies that seeking the correlation between air temperature indexes and the incidence of HFRS in mainland China during the period from January 2014 to February 2019. The appropriate adoption of geographical scale, data aggregation unit, the length of lag period and the length of incidence collection period should be considered when exploring the relationship between HFRS incidence and meteorological factors such as air temperature. Further investigation is warranted to detect the thresholds of meteorological factors for the HFRS early warning purposes, to measure the duration of lagged effects and determine the timing of maximum effects for reducing the effects of meteorological factors on HFRS via continuous interventions and to identify the vulnerable populations for target protection.

## Author summary

China has the largest number of hemorrhagic fever with renal syndrome (HFRS) cases in the world. With the acceleration of China’s urbanization process, especially in the process of rapid transition of China’s agriculture-related landscapes to urban landscapes, the dual role of climate change and environmental change has led to a leap in the epidemic area range of HFRS. Exploring or clarifying the relationship between HFRS epidemic and those environmental factors may help to grasp the spread and epidemic pattern of HFRS and then the pattern could serve as the partial basis of accurate HFRS incidence prediction and the corresponding allocation of public health resources. The present systematic review first described the heterogeneity of geographical scale, data aggregation unit and study period chosen in the ecological studies that seeking the correlation between air temperature indexes and incidence of HFRS in mainland China during the period from January 2014 to February 2019. Raising the awareness of the appropriate adoption of geographical scale, data aggregation unit, the length of lag period and the length of incidence collection period is of great importance when exploring the relationship between HFRS incidence and meteorological factors such as air temperature.

## Introduction

Hemorrhagic fever with renal syndrome (HFRS) is a rodent-associated zoonosis and also a legally mandated notifiable disease in China [1,2]. China has the largest number of HFRS cases in the world [3,4]. After the epidemic of HFRS in mainland China in the 1980s, the HFRS incidence fluctuated periodically with a cycle of about 5 to 10 years, but overall with a descending trend. In the 1990s, the annual number of HFRS cases reported was between 40,000 to 60,000 cases [5]. Since the beginning of the 21^st^ century, the HFRS incidence has continued to decline in mainland China, and after reaching the lowest level in nearly 20 years in 2009, it has gradually increased [6]. In the past three years, the number of HFRS incident cases was maintained at around 11,000 cases per year [7].

Climate and environmental changes could affect the reservoir ecology and dynamics of rodent carriers and hence trigger the spread of hantavirus transmission [5,8,9]. With the acceleration of China’s urbanization process, especially in the process of rapid transition of China’s agriculture-related landscapes to urban landscapes, the dual role of climate change and environmental change has led to a leap in the epidemic area range of HFRS. Exploring or clarifying the relationship between HFRS epidemic and those environmental factors may help to grasp the spread and epidemic pattern of HFRS and then the pattern could serve as the partial basis of accurate HFRS incidence prediction and corresponding health care resources allocation.

Due to the above-mentioned background and consideration, a comprehensive and in-depth collection, collation and analysis of data related to the epidemic status of HFRS is needed and this has become the consensus of researchers in the field of HFRS surveillance and prevention. Since the last century, an extensive literature has accumulated on the association between global climate change and infectious diseases. The involved meteorological factors included seasonal and climate change, greenhouse effect, tropical climate, ultraviolet light and etc., while in the field of HFRS prevention and control, the most studied meteorological factor was air temperature [10,11].

The choice of appropriate statistical methods according to the type of HFRS epidemic area, the availability, fineness and data distribution characteristics of historical data such as epidemic situation or meteorological data in various regions is being another challenge. The researchers adopted various statistical methods to explore the epidemiological links between HFRS incidence and air temperature, at different spatial and temporal scale and among those studies, the length of lag period also varied.

Hence, to characterize the variety of data aggregation scale and statistical methods adopted for the epidemiological links between the HFRS epidemic status and air temperature, we conducted the present systematic literature review for a broader appreciation. In this review, we assessed the current landscape of these studies in terms of user preferences, information needs of HFRS incidence data and air temperature data, and the considerations in the adoption of scale. Due to the challenges of rapidly changing situation of integrating and analyzing data, the review placed the emphasis on publication within the recent five years.

## Materials and methods

### Study selection

A systematic search in PubMed (www.ncbi.nlm.nih.gov/pubmed), Web of Science, Chinese National Knowledge Infrastructure (www.cnki.net), Wanfang Data (www.wanfangdata.com.cn) and VIP Info (www.vipinfo.com.cn) was performed to collect the publications related to the correlations between HFRS incidence and air temperature in mainland China. Data from Jan 1^st^, 2014 to February 28^th^, 2019 was retrieved by means of the keyword ‘hemorrhagic fever with renal syndrome’ in combination with the keywords ‘HFRS’, ‘epidemic hemorrhagic fever’, temperature’, ‘climate’, ‘meteorological factor’ and ‘China’ in both English and Chinese. References cited in the retrieved articles were also evaluated to maximize article recovery. The last search was conducted on March 20^th^, 2019.

### Inclusion and exclusion criteria

Eligible studies had to meet all the following six criteria: (i) they provided the data of the incidence of HFRS in mainland China; (ii) they provided the type of temperature indexes; (iii) they indicated the underlying geographical scale information, temporal data aggregation unit, and the data sources; (iv) they provided the statistical analysis method that had been used; (v) from peer-reviewed journals or dissertation; (vi) the time range for the inclusion of data exceeded two consecutive calendar years. Therefore, epidemiological studies providing HFRS incidence data only, or studies outside the boundaries of mainland China were excluded, review articles were also excluded. And when the studies were duplicately reported, the publication with larger sample size or more detailed data were included.

### Data extraction

As for each included study, the following information was extracted according to a self-designed data extraction form: first author and publication year, location and geographical scale (national, provincial, municipal and county/local-level), type of HFRS incidence data (incidence or number of HFRS incident cases), study period chosen, the air temperature indexes involved (average, maximum, minimum), temporal data aggregation unit (annual, seasonal, monthly, weekly and daily), the statistical method adopted, lag time considered, the correlation between HFRS incidence and air temperature that each included study concluded.

To ensure the reliability, two investigators (XHB and CP) independently screened each publication and the literature screening process was checked by a third reviewer (TJ). Two investigators (CP and TJ) independently summarized the data before discussing the results together, and all the discrepancies resolved by the principal investigator (PG).

### Risk assessment of study bias

According to the PRISMA Statement (S1 Table) [12], STROBE checklist [13], the bibliometric review by Dufault et al. [14] and the systematic review by Betran et al. [15], a self-designed quality assessment item list (S2 Table) was adopted to evaluate the quality of the included ecological studies. The risk of bias in the included ecological studies was evaluated with a total of ten risk-biased items regarding external validity (items 1 to 4 assessed the domain of selection) and internal validity (items 5 to 9 assessed the domain of measurement bias and bias of extrapolation or interpretation, and item 10 assessed the bias related to the funding). Two investigators (CP and ZMH) negotiated and completed the quality assessment. For the disagreements, the decision was made by the principal investigator (PG). After the data was extracted and double-checked, articles containing the study period, geographical location, type of HFRS incidence data, air temperature indexes involved, temporal data aggregation unit, description of statistical method adopted, correlation concluded between HFRS incidence and air temperature were considered to be qualified, and all the articles included in this systematic review met these quality requirements.

At the end of the overall risk assessment of study bias, according to the previously proposed criteria [16], studies with a “No” score ≤3 were classified as low risk, studies with a “No” score 4–6 were classified as moderate risk and studies with a “No” score ≥7 were classified as high risk. Studies with overall low and moderate risk of study bias were included in this present systematic review.

## Results

### Data acquisition and characteristics of the included studies

**Fig 1:**
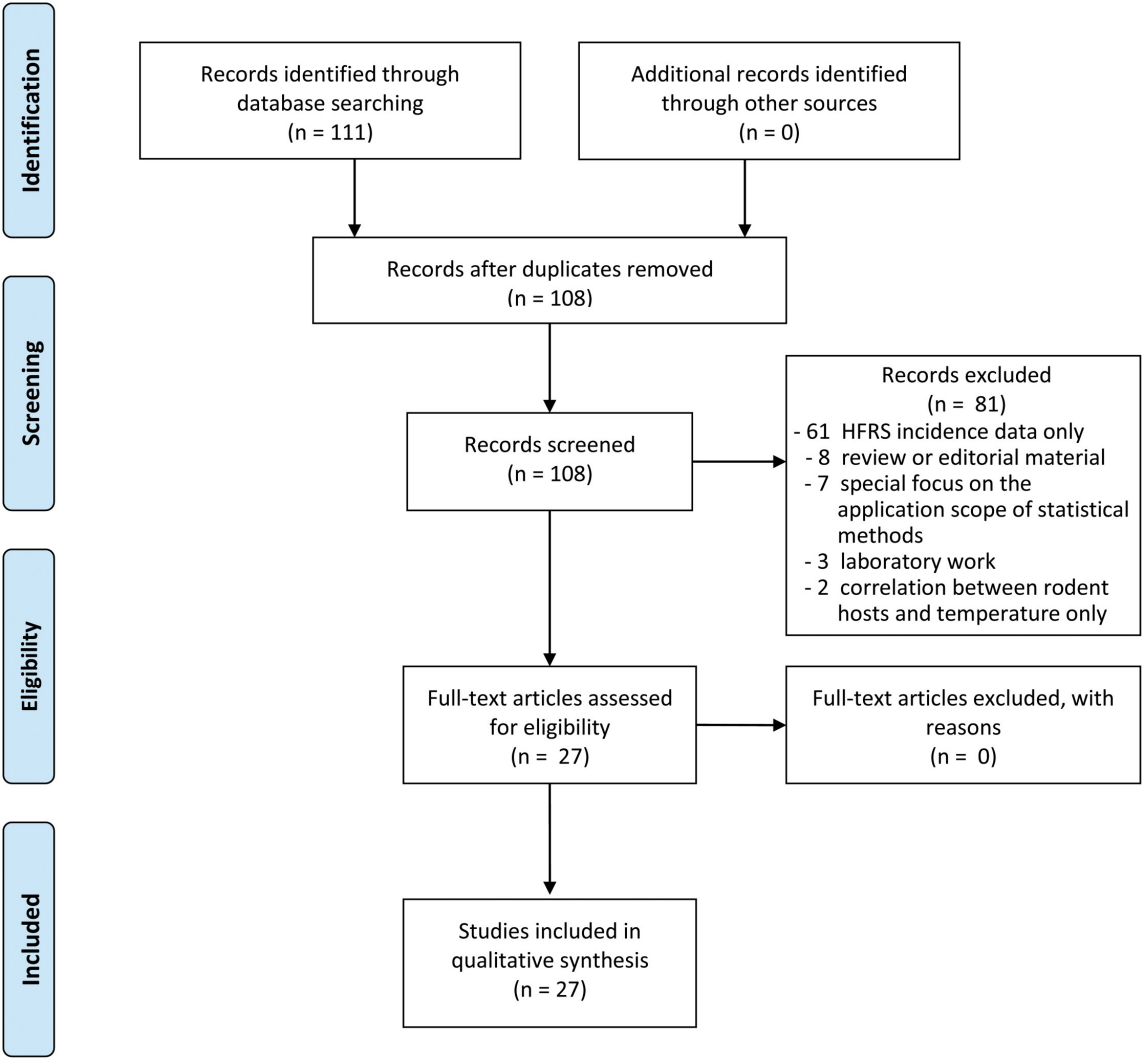
The preferred reporting items for systematic reviews and meta-analyses flow diagram.

A total of 111 articles related to the topic that published between Jan 1^st^, 2014 and Feb 28^th^, 2019 were identified, including 62 publications in Chinese and 49 publications in English. Three duplicated articles were subsequently removed, after intensive reading the titles, abstracts and full-texts of these article, 81 publications were also excluded. Thus, 27 publications (18 in Chinese and nine in English) were finally included in the systematic review, and among them 21 studies were with low risk of study bias and six studies were with moderate risk of study bias. The literature selection process is shown in Fig 1 and the PRISMA checklist is provided in S1 Table, the risk bias and assessment results are provided in S2 Table.

Among the 27 publications included, there were 22 journal articles and five dissertations; Those 22 journal articles scattered over 17 kinds of journals, with the journal ‘PLoS Neglected Tropical Diseases’ was identified as the most active journal about the topic during the study period. These 17 journals could be grouped into two categories, public health (thirteen kinds of journals), and natural sciences, including environmental sciences (four kinds of journals).

Among the included 27 studies, one study analyzed the HFRS data at the national level which included the data from 31 provinces, autonomous regions and municipalities in mainland China, and the remaining 26 studies involved 12 provinces and autonomous regions. Studies from Shandong Province accounted for 37.0% of all the included studies. Five studies collected HFRS data at provincial level, 17 studies collected HFRS data at municipal level, and four studies collected HFRS data at county (local) level. The study period of the data nested in the included studies ranged from 3 years to 54 years with a median of 10 years, as shown in Table 1.

**Table 1:** Characteristics of the included studies on the topic of air temperature and HFRS activity in mainland China, Jan 2014-Feb 2019.

Included studies	Geographical scale (survey area) & period	Type of HFRS data and temporal data aggregation unit	Temperature indexes (lagged time considered) and temporal data aggregation unit	Statistical methods	Major results regarding the correlation between air temperature and HFRS activity	Background information regarding the HFRS peak incidence
Bai, 2015 [17]	Provincial level (Chongqing), 1997–2008	Number of HFRS cases, monthly	T_ave_ (0 to 5 months), monthly	Poisson regression model	Negative correlation	Peaked in April, June, December. HFRS incidence was decreasing between 1997 and 2008
Cao, 2015 [18]	Municipal level (Changchun city in Jilin province), 1959–2012	Annual: incidence and number of HFRS cases; monthly: incidence and number of HFRS	T_ave_ (lagged effect not indicated), daily	Spearman correlation analysis	Positive correlation from late July to early September	Peaked in 1974–1989 and 1999–2006
Chen, 2016 [19]	Municipal level (Guangzhou city in Guangdong province), 2011–2014	Number of HFRS cases, daily	T_ave_ (lagged effect considered, maximum lag length not indicated), daily	Spearman correlation analysis and stepwise multivariate analysis	Negative correlation, when the daily average temperature was 15.2 °C, lagged by 10 days with the greatest risk	Peaked in May to September
Chen, 2016 [20]	Municipal level (Yancheng city in Jiangsu province), 2005–2014	Number of HFRS cases (monthly); HFRS incidence (annual)	T_max_, T_min_, T_ave_ (lagged effect considered, maximum lag length not indicated), monthly	Pearson correlation analysis	Negative correlation	Peaked in winter
Cheng, 2014 [21]	Provincial level (Jiangsu province), 2000–2009	Number of HFRS cases, monthly	T_max_, T_min_, T_ave_ (0–1 month), monthly	Correlation analysis	No strong correlation; in a few years, negative correlation with T_min_	Spring and summer (May-July, relatively small peak), autumn and winter (October-January, relatively big peak)
Cong, 2014 [22]	Municipal level (Gannan Tibetan Autonomous Prefecture in Gansu Province), 1985–2005	Number of HFRS cases, monthly and seasonal	T_ave_ (lagged effect considered, maximum lag length not indicated), monthly	Correlation analysis	Negative correlation for monthly number of HFRS cases, no correlation for seasonal number of HFRS cases	Peaked in autumn and winter (mainly in December)
Guo, 2017 [23]	Municipal level (Weifang city in Shandong province), 1974–2016	Number of HFRS cases, monthly	T_ave_ (0–6 months), monthly	Spearman correlation analysis and multiple linear regression analysis	Inverted U-shaped, 12°C as the apex; correlation was positive when T_ave_ <12 °C and negative when T_ave_>12 °C	Peaked mainly in autumn and winter
He, 2018 [24]	County-level (Raohe county and Mishan county in Heilongjiang province and Chang’an county and Hu county in Shaanxi province), 2000–2012	Number of HFRS cases, monthly	T_max_ (0–5 months), monthly	SARIMAX	No correlation found	-
Lao, 2018 [25]	Municipal level (Huludao City, Liaoning province), 2005–2012	Number of HFRS cases, monthly	T_ave_ (0–2 months), monthly	GAM based on Poisson distribution	Negative correlation and different effects on people with different characteristics	Peaked in winter and spring
Li, 2016 [26]	County level (counties under Jining’s jurisdiction in Shandong province, 2004–2014	Number of HFRS cases, annual and monthly	T_ave_ (lagged effect not indicated), annual and monthly	Cochran-Armitage trend test analysis, Spearman rank correlation analysis	Negative correlation between monthly HFRS cases and T_ave_, few correlations between annual HFRS cases and T_ave_	Peaked from January to May, from November and December
Li, 2016 [27]	County level (counties in Shandong Province), 1974–2012	Number of HFRS cases, monthly	Monthly T_ave_ in spring and autumn); monthly T_max_ in summer; monthly T_min_ in winter (0–6 months)	Conditional logistic regression (case crossover design)	Positive correlation with T_ave_ in spring	Peaked in winter (October- December), and in spring and summer (April-June). Two HFRS incidence peaks occurred in 1986 and 1995
Li, 2014 [28]	National level 31 provinces, autonomous regions and municipalities of China, 2005–2012	HFRS incidence, annual	T_ave_ (lagged effect not indicated), annual	Pearson correlation analysis, GWR Model	Negative correlation in 2005–2007; effect of temperature more pronounced in the northeast than in the southwest region	-
Lin, 2014 [29]	County-level (Jiaonan county in Shandong province), 2006–2011	Number of HFRS cases, daily	T_ave_ (0–14 days), daily	GAM (penalized smooth spline method)	Inverted U-shaped, 17°C as the apex; correlation was positive when T_ave_<17 °C (lag 0–3 days, 7–11 days) and negative when T_ave_>17 °C (lag 0–14 days)	Peaked in autumn and winter (October-December), and smaller peak in May and June
Tao, 2015 [30]	Municipal level (Qinhuangdao city in Hebei province), 2005–2012	Number of HFRS cases, annual and monthly	T_ave_ (annual); T_ave_ (lagged effect not indicated, monthly)	Linear correlation analysis; Stepwise regression analysis	Negative correlation between the number of monthly HFRS cases and T_ave_ and no correlation between the number of annual HFRS cases with T_ave_	Big peak in 2005, small peak in 2012
Tian, 2015 [31]	Municipal level (Xi’an city in Shaanxi province), 2005–2012	HFRS incidence, monthly	T_ave_ (0–6 months), daily	Time series wavelet analysis, Bayesian time-series Poisson adjusted model, correlation analysis	Positive correlation (4 months lagged)	Peaked from October-December
Wang, 2018 [32]	Municipal level (Qingdao city, Shandong province), 2007–2015	Number of HFRS cases, monthly	T_ave_ (0–6 months), monthly	Correlation analysis and GAM	Positive correlation (2, 3, 4 and 5 months lag)	Peaked from late autumn to spring of the following year
Wang, 2015 [33]	Municipal level (Changsha city in Hunan province), 2004–2014	Number of HFRS cases, monthly	T_max_, T_min_, T_ave_ (0–3 months), monthly	Time-delayed correlation analysis, ridge regression	Positive correlation with T_min_ (one month lag).	Peaked mainly from November-January, and smaller peak in April-June
Wei, 2014 [34]	Municipal level (Linyi city in Shandong province), 2007–2012	Number of HFRS cases, monthly	T_ave_ (lagged effect not indicated), monthly	Spearman correlation analysis, log-regression curve fitting	Weak correlation between HFRS incidence and temperature.	Peaked autumn and winter (October-December)
Wei, 2018 [35]	Municipal level (Guangzhou city in Guangdong province), 2006–2015	Number of HFRS cases, monthly	T_ave_, T_max_, T_min_ (0–3 months), daily data collected and thus monthly data calculated	Negative binomial multivariable regression	Negative correlation with T_ave_ (0–3 months lag)	Peaked from February to May
Wu, 2014 [36]	Provincial level (Liaoning province), 2005–2007	HFRS incidence, annual	T_ave_ (lagged effect not indicated), annual	Spearman correlation analysis	Negative correlation	Peaked in Winter and spring (November- January, March-May)
Xiang, 2018 [11]	Municipal level (19 cities under the jurisdiction of Anhui, Heilongjiang and Liaoning provinces), 2005–2014	Number of HFRS cases, weekly	T_max_ (0–34 weeks), weekly	GEE and multivariate; random-effects meta-regression models	Positively correlation with T_max_ in 18 cities	Peaked in autumn and winter; for Heilongjiang and Liaoning provinces, peaks in spring and autumn
Xiao, 2014 [37]	Municipal level (Chenzhou city in Hunan province, 2006–2010	HFRS incidence, monthly	T_ave_ (0–6 months), monthly	Cross correlation analysis, PDL model	Positive correlation (4–5 months lagged)	Peaked from May-June, November-January (the following year)
Xu, 2018 [38]	Municipal level (Weifang city in Shandong province), 2005–2015	Number of HFRS cases, daily	T_ave_ (0–30 days), daily	DLNM	Negative correlation; HFRS risk was higher when the temperature ranged between 0 and 15 °C.	Peaked from March to May and October to December
Xu, 2018 [39]	Municipal level (Qingdao city in Shandong province), 2007–2013	Number of HFRS cases, daily	T_ave_, T_max_, T_min_ (0–30 days), daily	DLNM	Negative correlation with T_ave_ with lagged effect	Peaked during April-June and October-January (the following year)
Xu, 2018 [40]	Provincial level (Shandong province), 2007–2012	Number of HFRS cases, daily	T_ave_, T_max_, T_min_ (0–30 days), daily	Spearman correlation analysis and Quasi-Poisson regression with DLNM	Negative correlation	Peaked in March-June and October-December
Yu, 2016 [41]	Provincial level (Guangdong province), 2004–2014	Number of HFRS cases, monthly	T_ave_, T_min_ (0–4 months), monthly	Cross-correlation analysis, time series ARIMA model	Negative correlation with T_ave_ and T_min_ (2-month lagged effect)	Peaked during March-June; December-January (the following year)
Zhang, 2017 [42]	Municipal level (Anqiu city in Shandong province), 2000–2014	Number of HFRS cases, monthly	T_ave_ (0–2 months), monthly	Spearman correlation analysis and multiple linear regression analysis	Negative correlation (0–2 months)	-

Abbreviations: T_ave_, average air temperature; T_max_, average maximum air temperature; T_min_, average minimum air temperature; ARIMA, Autoregressive Integrated Moving Average model; DLNM, Distributed lag nonlinear model; GAM, Generalized additive model; GEE, Generalized estimating equation models; GWR, Geographically weighted regression model; PDL, polynomial distributed lag model; SARIMAX, Seasonal autoregressive integrated moving average model with exogenous variables

Regarding the temporal unit of data aggregation, in the included studies, fifteen studies were based on monthly HFRS incidence or the number of monthly incident HFRS cases, five studies were based on the number of daily reported HFRS cases, two studies based on annual HFRS incidence, four studies based on both monthly and annual HFRS incidence, and one study was based on the number of weekly reported HFRS cases. As for the corresponding air temperature indicators, seven studies adopted all the three indicators of average air temperature, average maximum air temperature and average minimum air temperature, and the rest of the studies were based on either average air temperature only or average maximum air temperature only. Twenty-one studies considered the lagged effect of air temperature factors on the HFRS activity and the longest lag period considered in the included studies was 34 weeks.

With regards to the statistical methods adopted by the researchers to explore the correlation between air temperature and HFRS incidence, Spearman correlation analysis, Pearson correlation analysis, generalized additive model, seasonal differential autoregressive moving average model, negative binomial multivariate regression analysis, distributed lag nonlinear model conditions, conditional logistic regression analysis and wavelet analysis have been indicated in the included studies, as shown in Table 1.

### Correlation between HFRS activity and air temperature varied widely

The associations observed at one scale were not present at another one. Fifteen studies indicated the negative correlation between air temperature and HFRS activity, while seven studies found the positive correlation between air temperature and HFRS activity. There were also two studies that defined a certain temperature as the dividing point between HFRS activity and air temperature, the correlation curve was inverted ‘U-shaped’ [23,29]. Also, three studies did not find statistically significant associations between HFRS activity and air temperature.

### Effect of temperature on the HFRS epidemic was seasonal

Because the current China’s National Notifiable Infectious Disease Reporting system (NIDR) is still unable to distinguish the type of hantaviruses infected by HFRS patients, the direction and magnitude of the effects of temperature on HFRS activity in different seasons were also inconsistent. The study based on the monthly temperature index at county scale in Shandong Province found that the increase of average air temperature in spring was the risk factor of SEOV-type HFRS outbreak, but similar results could not be found in other seasons and in the HTNV-type HFRS [27]. The study from Changsha City indicated that HFRS incidence was highly correlated with the previous air temperature [33]. The study from Changchun City, Jilin Province [18] and Changsha City [33] found that HFRS was prone to outbreaks and epidemics during relatively dry, high temperature and low wind conditions from late July to late September. The study in Guangdong Province [41] indicated that rainy or cloudy conditions in the previous season might contribute to the growth of the number of HFRS cases. The study in Guangzhou City found that the incidence of HFRS was negatively correlated with the daily average air temperature, and when the average daily temperature was 15.2 °C, the relative risk reached highest with the lag period of ten days [19].

### Effect of air temperature on the HFRS activity varied among different populations

In addition to collecting data on the incidence of HFRS in the whole population, a certain study also collected data on HFRS incidence (age, gender, occupation, etc.) from different populations based on the national legal infectious disease surveillance system. The study in Huludao City, Liaoning province found that HFRS activity in the population aged 35–59 years was significantly affected by air temperature, but this phenomenon could not be found in the population of other age groups [25]; the authors of this Huludao study attributed the phenomenon to the increased virus exposure in agricultural labor work in the middle-aged population. This study in Huludao also indicated that the correlations between HFRS activity and air temperature were statistically significant in both males and females, while with different length of period, two months in the male and no lag in the female population [25].

## Discussion

It is clear from the major part of the included studies that air temperatures are indirectly associated with HFRS activity, however, the temperature-HFRS association findings were inconsistent and location-dependent. Our systematic review indicated that the ecological effects of air temperature on HFRS incidence could be affected by the spatial or temporal scale of the data and also the study period involved, which might help to partly understand the contradicting observations in the included studies. The researchers need to consider or identify which temperature indicator and data aggregation unit are more appropriate to explain the correlations between HFRS incidence and air temperature at different geographical scales [43].

Seeing that the effect of air temperature on the HFRS activity varied among different populations [25], it is suggested that the demographic characteristics of the local target population should be considered when the correlation between meteorological factors, such as air temperature, and HFRS incidence is estimated or predicted to assist precise HFRS prevention and control.

The ecological correlation analysis is not only data-driven, but also technology-concentrated, the integration of the collection of high-quality HFRS incidence data and the multi-discipline development can open vast vistas for the correlation analysis techniques’ application in the field of infectious diseases epidemiology [44]. In the real world, only when the statistical methods that can be understood and adopted by the HFRS incidence data collectors and the users of infectious disease surveillance system, the relationship between HFRS incidence and meteorological factors could be better clarified. And then, these correlations could be possibly incorporated into the early warning, prevention and control of HFRS. According to the observed correlations between HFRS activity and air temperature, with special considerations of geographical scale, data aggregation unit, length of lag period and study period, appropriate statistical methods nested in the National Notifiable Infectious Disease Reporting system will be of great importance for the users.

The present systematic review might be improved if the following information could be considered in the future HFRS-related ecological studies. All the included studies in the present systematic review are descriptive, further exploratory analysis, explanatory analysis and statistical inference are needed. The distribution of Hantaan and Seoul type of HFRS was not available in the China’s surveillance system of the legally mandated notifiable infectious diseases. The data of HFRS vaccine coverage could not be obtained in the included studies, and HFRS vaccine coverage did affect the magnitude of HFRS incidence. Only one single included study investigated the correlation between air temperatures and HFRS incidence stratified by age group, thus the characteristic of the HFRS vulnerable population that was most affected by air temperature could not be obtained. Besides the different data scale and the differences in the hantaviruses type of HFRS, the factors relating to the dynamics of the rodent hosts and human activities, such as urbanization indicators, should be considered when understanding the results from these ecological studies, given the fact that China is a topographically heterogeneous country. It should also be emphasized that air temperature as an isolated indicator that cannot explain the HFRS incidence fully, confounding factors should always be considered. Caution should be used when studying the associations between HFRS activity and the isolated or the combination of meteorological variables, because of the possible multicollinearity. Therefore, meteorological factors and the impact of climate changes on the pathogenesis of HFRS still need to be further deepened, especially in the process of rapid transition of China’s agriculture-related landscapes to urban landscapes [45].

In summary, the present systematic review first described the heterogeneity of geographical scale, data aggregation unit and study period chosen in the ecological studies that seeking the correlation between air temperature indexes and the incidence of HFRS in mainland China during the period from January 2014 to February 2019. The appropriate adoption of geographical scale, data aggregation unit, the length of lag period and the length of incidence collection period should be considered when exploring the relationship between HFRS incidence and meteorological factors such as air temperature. Further investigation is warranted to detect the thresholds of meteorological factors for the HFRS early warning purposes, to measure the duration of lagged effects and determine the timing of maximum effects for reducing the effects of meteorological factors on HFRS via continuous interventions and to identify the vulnerable populations for target protection.

## Supporting information

S1 Table PRISMA checklist. (DOC)

S2 Table Risk of bias assessment. (XLSX)
